# Benign giant pilomatricoma with associated hypercalcemia: a case report

**DOI:** 10.1093/jscr/rjag001

**Published:** 2026-02-27

**Authors:** Colton Cox, Kyren Maynard, Babe Westlake D O

**Affiliations:** MD Program, University of Nevada Reno School of Medicine, 1664 North Virginia Street, Reno, NV 89557, United States; MD Program, University of Nevada Reno School of Medicine, 1664 North Virginia Street, Reno, NV 89557, United States; Department of Orthopedic Oncology, Renown Health, 1155 Mill Street, Reno, NV 89502, United States

**Keywords:** pilomatricoma, hypercalcemia, soft tissue mass

## Abstract

The patient presented to the emergency department with nausea and vomiting with a large, rapidly growing soft tissue mass on her left shoulder diagnosed as a giant pilomatricoma. Pilomatricomas are tumours derived from hair follicles that are typically benign and rarely cause systemic symptoms. However, this patient exhibited hypercalcemia and elevated parathyroid hormone-related peptide (PTHrP) levels, suggesting PTHrP secretion by the tumour. Surgical resection normalized the patient's calcium and PTHrP levels. This case demonstrates that tumours such as pilomatricomas can rarely secrete PTHrP and cause hypercalcemia, a characteristic not typically associated with benign growths. It emphasizes the importance of considering benign tumours in the differential diagnosis of hypercalcemia.

## Introduction

Pilomatricomas are uncommon tumours originating from hair follicles that are usually benign, with the highest incidence in the pediatric population. These tumours develop typically in hair-bearing areas, frequently on the head and neck. Females exhibit a higher predisposition to pilomatricoma development compared to males. The reported frequency of pilomatricoma ranges from ‘0.001% to 0.0031% of all dermatopathology specimens and 20% of all pilar lesions’ [1]. Initially presenting as small nodules, these tumours can exhibit rapid growth over several months [2] and are thought to be associated with symptoms local to the tumour. We report a case of giant pilomatricoma-induced hypercalcemia causing systemic symptoms that are not typically associated with benign tumours.

## Case report

We present the case of female in her twenties who sought care for nausea, vomiting, and body aches. In the emergency department, she was found to have a large soft tissue mass on her left anterior shoulder concerning for a sarcoma. Laboratory studies revealed acute hypercalcemia with a serum calcium of 13.3 mg/dl (reference range 8.5–10.5 mg/dl), requiring admission.

**Figure 1:**
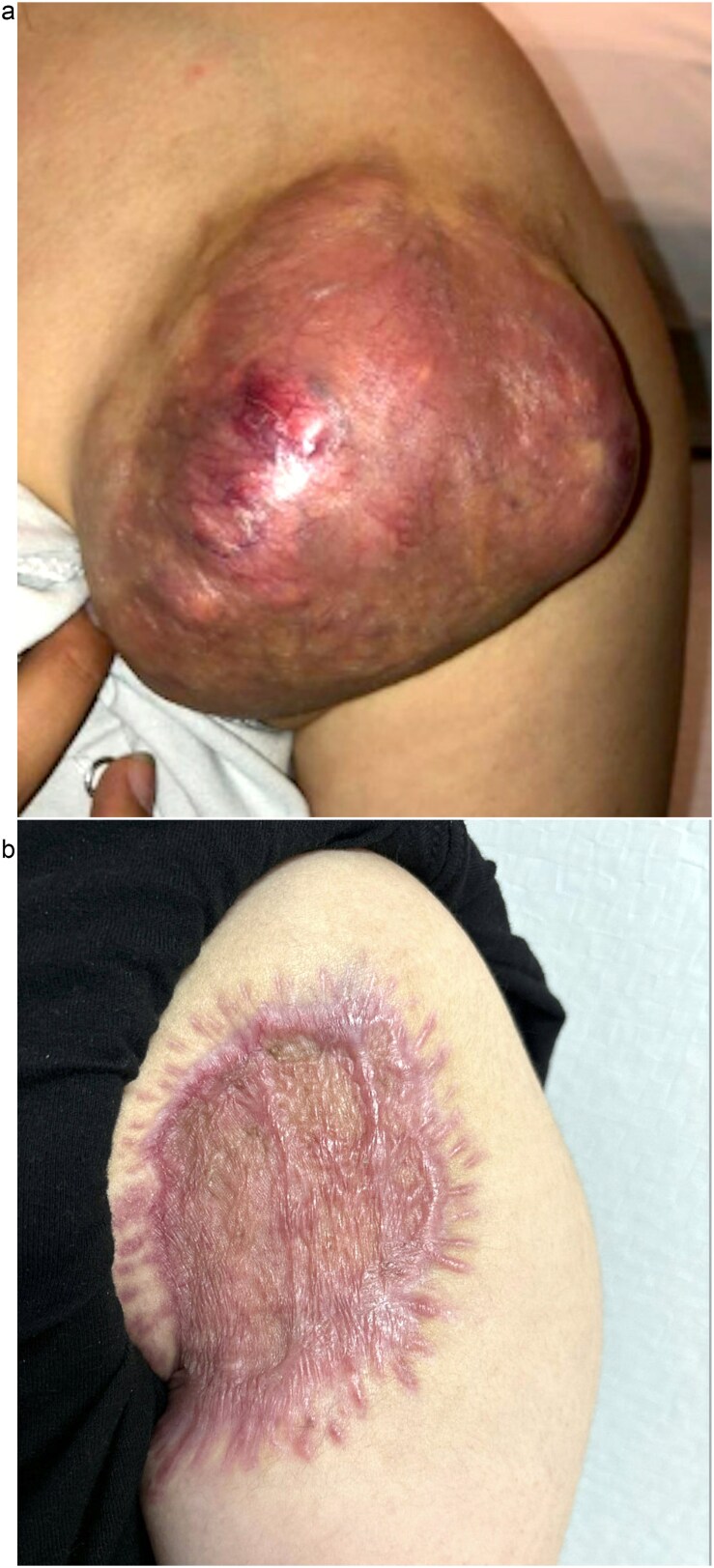
Pre-operative image (a) and image at follow-up with a healing skin graft (b) of the left anterior shoulder.

The patient reported that the mass had been present for ~3 years. An initial surgical consultation identified it as a cyst and no treatment was pursued. Over the past year, however, the mass at least doubled in size, with edematous, thinned overlying skin but no ulceration (Fig. 1a). Additionally, she complained of ipsilateral breast and rib tenderness, persistent nausea, vomiting, fatigue, body aches, and right lower extremity pain spanning several months. Her past medical history was unremarkable, with no chronic conditions, allergies, surgeries, or tobacco use.

**Figure 2:**
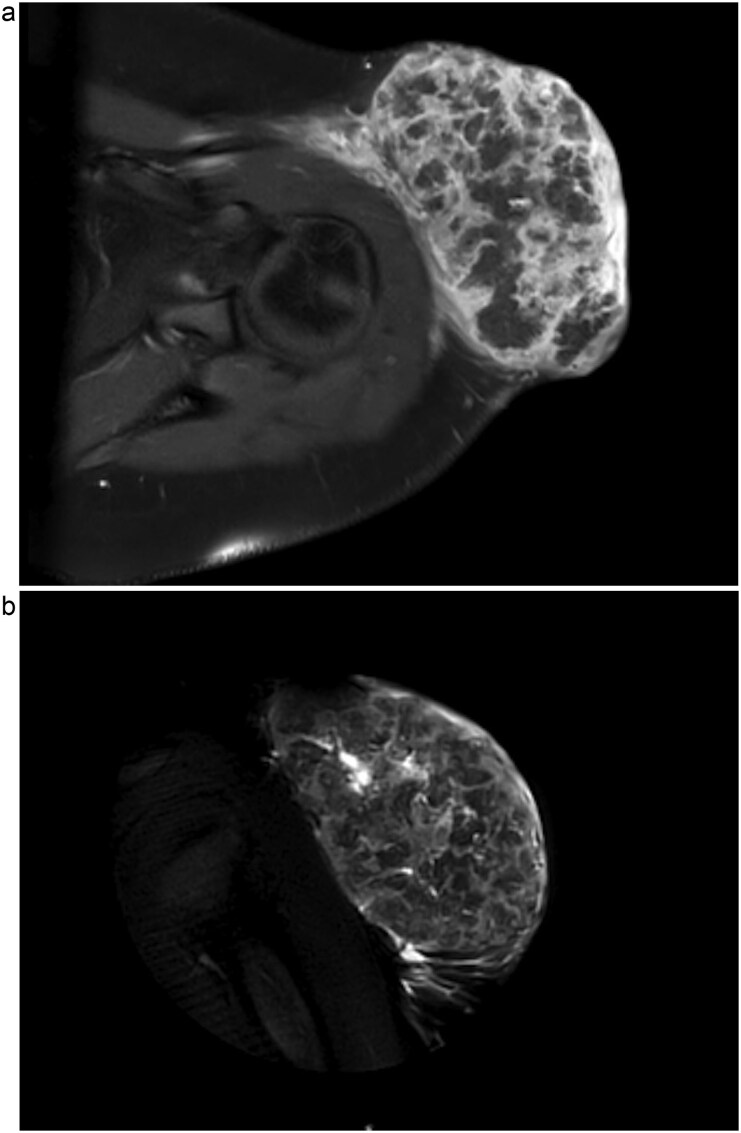
1.5 Tesla MRI with axial view (a) and coronal view (b) showing a 10.8 × 6.7 × 7.4 cm left anterolateral shoulder soft tissue mass. This is suspicious for malignancy, most likely sarcoma, due to lack of a soft tissue plane between the tumour and the deep deltoid musculature as well as enlarged axillary lymph nodes (not shown).

**Figure 3:**
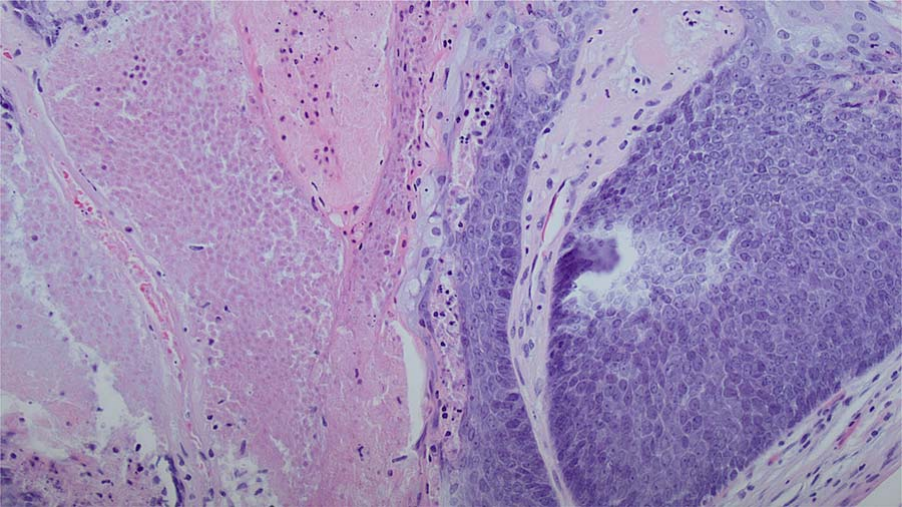
A dual cell population of basaloid cells and anucleate ghost cells with associated necrosis. This is a classic finding for a benign pilomatricoma of this size. Magnification of 20× and stained with haematoxylin and eosin.

**Figure 4:**
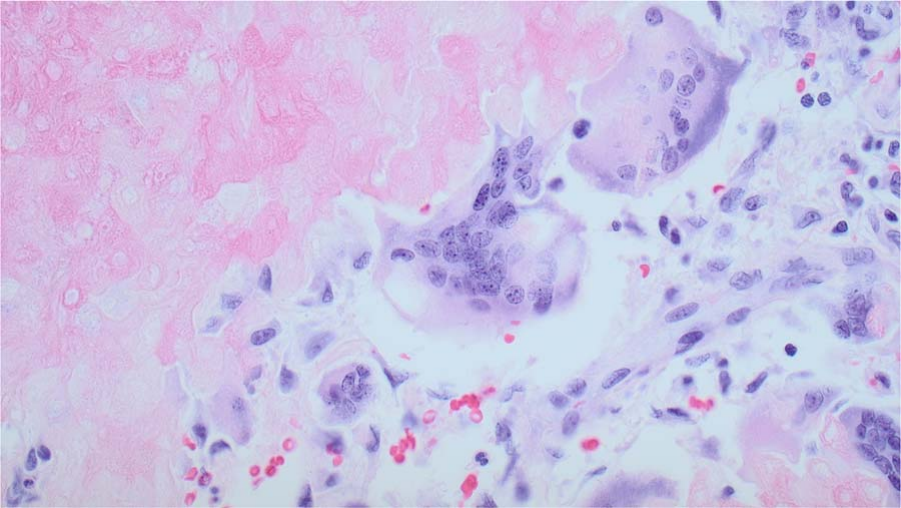
A multinucleated giant cell within the pilomatricoma. Derived from macrophages, this likely developed as an immune response to the necrotic ghost cells. Pilomatricomas are dermal tumours derived from hair follicle matrix cells, which undergo abrupt keratinization leading to the formation of ghost cells. These ghost cells contain degenerated nuclear and cellular material that stimulate macrophages and their fusion into multinucleated giant cells. Magnification of 20x and stained with haematoxylin and eosin.

Upon admission, computed tomography (CT) and magnetic resonance imaging (MRI) revealed a 10.8 × 6.7 × 7.4 cm soft tissue mass overlying the left anterior shoulder and axillary lymphadenopathy. The MRI noted lack of a soft tissue plane between the mass and deltoid muscle, indicating possible invasion of the muscle tissue (Fig. 2a and b). The radiologist expressed concern for malignancy. Core needle biopsy of the mass described it as a cystic, partially solid mass comprised of necrotic ghost cells, multinucleated giant cells, basaloid epithelium, and squamous epithelium. These findings, accompanied by the tumour’s size, were sufficient for a diagnosis of giant pilomatricoma (Figs 3 and 4). A concurrent axillary lymph node biopsy was negative for lymphoproliferative disorder and malignancy. The PTHrP level was considerably elevated at 61.0 pmol/L (reference range 0–3.4 pmol/L). We suspected the pilomatricoma was secreting PTHrP and causing her hypercalcemia, a rare but documented occurrence in the literature [3].

She received IV fluids and later pamidronate to stabilize calcium levels. Wide local excision was then performed. Pathology confirmed the tumour to be excised with negative margins. A temporary wound vacuum-assisted closure (VAC) was placed over the extensive surgical defect. The specimen revealed the mass to be compressing the surrounding soft tissue without invasion, leading the pathologist to classify the pilomatricoma as benign. Although necrosis within tumours is a finding typically associated with malignancy, it was believed to be caused by the tumour outgrowing its vascular supply. The patient’s serum calcium levels normalized following the surgery and she later returned to the operating room for split-thickness skin grafting from her left thigh to the left shoulder surgical wound. At follow-up, the wound VAC was removed and her surgical sites were healing without evidence of complication or recurrence (Fig. 1b).

## Discussion

This report describes a rare case of a benign giant pilomatricoma secreting PTHrP and causing hypercalcemia, which resolved with tumour resection. The patient's presentation is scarcely documented in the medical literature.

A comprehensive literature search of the PubMed database identified 16 articles relevant to hypercalcemia associated with benign tumours, primarily consisting of case reports and case series. One other case of giant pilomatricoma causing hypercalcemia was found [3]. However, the case differed by presenting with purulent discharge and blood from the tumour, anemia, hypoalbuminemia, and no lymph node involvement. They also reported a much lower PTHrP level of 4.1 pmol/L in comparison to the 61.0 pmol/L our patient had, which is surprising considering the larger relative size of their reported tumour (20 × 9 × 10 cm vs. 10.8 × 6.7 × 7.4 cm). This discrepancy suggests tumour size may not correlate with PTHrP secretion. Thus, even small benign tumours should be considered in the differential diagnosis for hypercalcemia.

According to the literature search, hypercalcemia related to benign tumours is commonly caused by parathyroid gland neoplasms, resulting in hyperparathyroidism. Hypercalcemia due to benign tumours outside the parathyroid gland is extremely rare and more often reported with uterine or renal tumours, such as congenital mesoblastic nephroma [4], leiomyoma [5, 6], and pheochromocytoma [7] rather than a hair follicle.

Normalization of the elevated levels of PTHrP and calcium within the blood following tumour resection provides compelling evidence for the causal relationship between the hypercalcemia and pilomatricoma. PTHrP has a biochemical mechanism similar to endogenous parathyroid hormone (PTH), increasing calcium absorption and stimulating osteoclasts to release calcium from bone. Hypercalcemia is known to be associated with malignant tumours, with this case being a highly unexpected exception and is the primary novel finding within this report. The exact mechanism of PTHrP secretion by pilomatricoma remains unclear, underscoring the need for further study.

While this patient presented with hypercalcemia-induced nausea, vomiting, and body aches, other presentations of hypercalcemia include kidney stones, constipation, polyuria, arrythmias, and depression. Acute hypercalcemia management includes fluid resuscitation, calcitonin and bisphosphonates. Patients with severe hypercalcemia needing emergent treatment may receive dialysis. Chronic hypercalcemia requires treatment of the underlying cause, such as parathyroidectomy [8]. When left untreated, hypercalcemia can cause kidney failure and decreased bone density seen in osteoporosis [9]. In this case, the patient underwent excision of the giant pilomatricoma before long-term complications arose. The early identification of the tumour as a possible cause of the hypercalcemia was somewhat due to its large size. Future cases of hypercalcemia may be associated with smaller benign neoplasms that act insidiously and without obvious cause. This case highlights the importance of having a wide differential when seeking to identify the reason for a patient’s hypercalcemia, including benign neoplasms.
